# Compression Dewatering Forming: A Rheology-Driven Approach to Produce Complex-Shaped Prefabricated Cement Products

**DOI:** 10.3390/ma18071607

**Published:** 2025-04-02

**Authors:** Chunlei Xia, Qianping Ran, Xiongfei Zhang, Xiaorong Wang

**Affiliations:** 1 Beijing Municipal Engineering Research Institute, Beijing 100037, China; xiachunlei2005@163.com (C.X.); xf251019@163.com (X.Z.); wangxr1104@163.com (X.W.); 2 School of Materials Science and Engineering, Southeast University, Nanjing 211189, China

**Keywords:** complex shapes, mortar, compression dewatering, compressive strength, porosity, rheological properties, formability

## Abstract

With the development of prefabricated buildings, complex-shaped cement products, represented by heating-type elevated floors, have appeared on the market. These cement products can only be produced by the pouring method, with low efficiency and poor precision. Among the existing processing methods for preparing cement products, compression dewatering offers the greatest ability to produce cement products with complex shapes. However, the pressed mixing material comprises a plastic fresh mortar, which inherently lacks fluidity, making it difficult to completely fill the cavity of the shaped mold. Few studies have been conducted on the experimental method and design ratios of mortar for the compression dewatering process in the industry, with no effective solution. To achieve the efficient production of complex-shaped cement products, this study explored the experimental method of testing the strength and flowability of mortar formed through compression dewatering as the forming process. Mortar ratios suitable for producing complex-shaped cement products using the compression dewatering process were determined, the relationship between material rheology and product forming performance was analyzed, and the influence of the compression process on the strength and micro-properties was studied. Finally, a cement-based heating-type elevated floor forming technology was developed, offering a novel approach for the efficient forming of complex-shaped cement products.

## 1. Introduction

**Figure 1:**
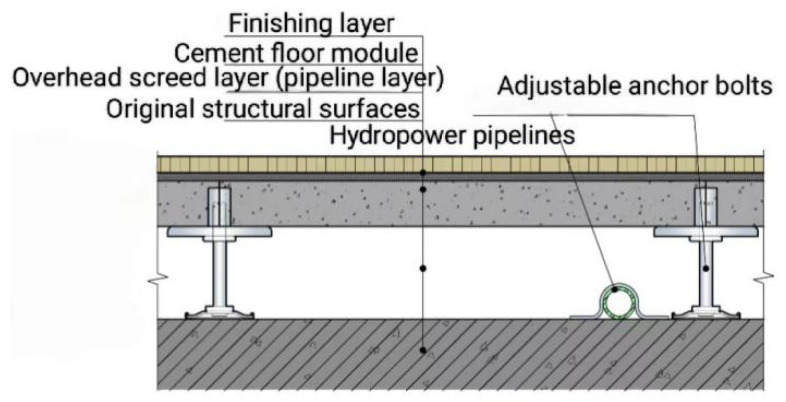
Schematic diagram showing the overhead floor structure of a heated composite panel module.

**Figure 2:**
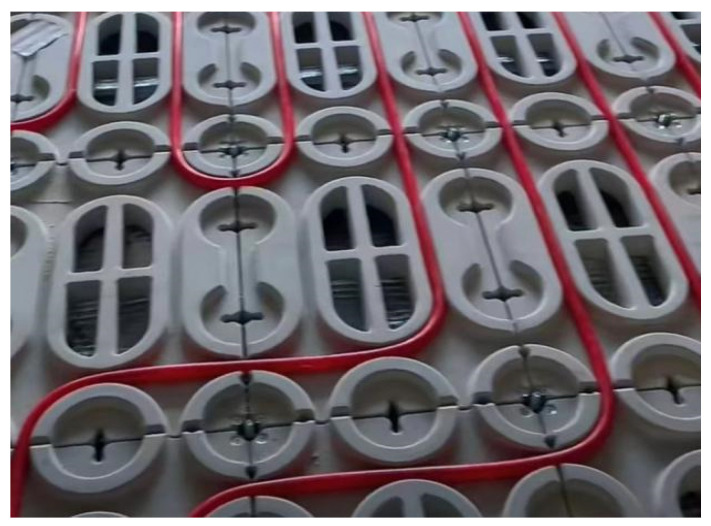
Complex raised floor shape.

**Figure 3:**
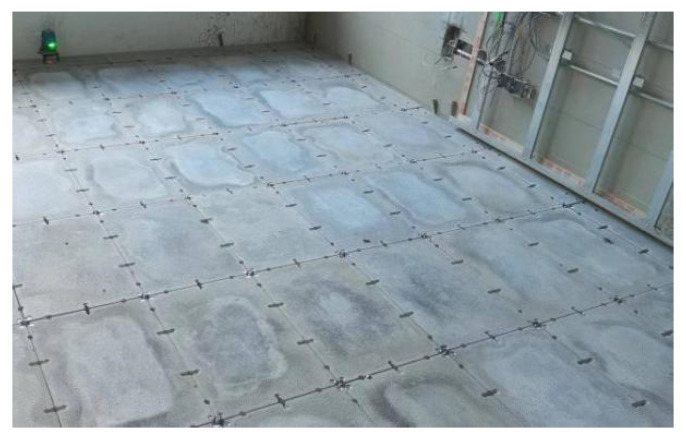
Flat raised flooring.

With the Chinese government actively promoting prefabricated buildings, the development of prefabricated decoration has accelerated significantly. By 2022, the area of prefabricated decoration in China reached 92 million m^2^ [1]. As an important part of the prefabricated decoration, prefabricated raised flooring [2] has been rapidly developed in recent years (Figure 1), including heating-type and functional-type raised floors. This has led to the emergence of cement-based raised floors with complex shapes, such as grooves (Figure 2), replacing the traditional simple flat slab design (Figure 3). These types of raised floors enable the integration of heating pipes into the raised floor, providing not only structural load-bearing capabilities but also heating functionality, as well as accommodating plumbing and electrical pipework simultaneously.

The primary forming methods for cement-based raised flooring include pouring and compression forming. Among these, compression forming has become the dominant production method for cement-based raised flooring due to its high degree of automation and high precision. Initially, dry vibration compression forming was employed. In this process, particles collide under high-frequency vibrations (3000~12,000 times/min), where dynamic friction replaces static friction between the particles, turning the fluid into fluid-like particles. These particles gradually compact under their own and external pressure to form dense billet [3,4]. While this method dominates pressed cement products because of its high efficiency and low cost, the resulting product quality is relatively low. To address these limitations and produce high-quality cement products, the compression dewatering forming process was developed. This process involves mixing mortar with water to achieve a plastic state, injecting the mixture into a mold equipped with dewatering functionality and then compressing the materials into shape using a high-pressure press. Excess water is then discharged, resulting in a dry and hardened product. Compression dewatering forming enhances the product strength and surface texture and supports different shapes. It is widely applied in the production of overhead flooring, cement tiles, and cement-based curbs [5,6] and finds use in the engineering of roads, harbors, and bridges [7,8,9].

Dry vibratory compression forming and compression dewatering processes are primarily suited for producing simple-shaped plates. For complex-shaped cement products, the inefficient pouring method is used. In-depth studies found that the key factor restricting the compression forming of complex-shaped cement products was the insufficient fluidity of the compressed mortar, making it difficult to completely fill the cavities of shaped molds. Experimental investigations revealed that materials employed in dry vibration compression forming exhibit limited fluidity, whereas those in compression dewatering processes can attain controlled fluidity characteristics under applied pressure. However, based on conventional ratios, this fluidity remains inadequate for complex-shaped cement products, often leading to issues, such as a lack of material. In addition, the experimental method for evaluating the compression dewatering forming of mortar remains underdeveloped, with a lack of effective experimental methods to study the flowability and compressive strength of compression dewatering forming mortar, restricting material-optimization efforts. Existing studies lack systematic optimization of rheological properties for pressed mortar, particularly in quantifying the synergy between mineral admixtures (fly ash, silica fume) and viscosity modifiers.

To achieve the efficient production of complex-shaped cement products, this study adopted compression dewatering as the forming process. Experimental methods suitable for evaluating the strength and fluidity of materials using this process were explored through a systematic investigation of critical factors, including water-migration patterns, porosity evolution, and their combined effects on compressive strength characteristics. This study also explored the effects of external admixtures, such as fly ash, silica fume, and additives, such as water and viscosity reducers, on the rheology and forming properties of mortar. The findings revealed how mortar rheology impacted the forming performance of pressed complex-shaped cement products. Based on these experiments, a novel and efficient process to produce complex-shaped cement products, denoted as the compression dewatering process, was developed. This method offers new solutions for the efficient production of complex-shaped cement products, including multifunctional prefabricated manhole covers, decorative elements, cement vignettes, and even cement furniture. The process significantly broadens the application potential of mortar, paving the way for their adoption in diverse and innovative applications. What is more, to address the scientific laws related to the rheology of mortar, this study employed a high-precision rheological evaluation method using an MCMR rheometer, overcoming the limitations of traditional standards (e.g., JGJ/T70-2009) for low-fluidity slurries. On the academic level, this study revealed the relationship between rheology and the formability of mortar, providing theoretical support for the suppressing production of complex-shaped cement products.

## 2. Materials and Production Process

### 2.1. Materials

**Figure 4:**
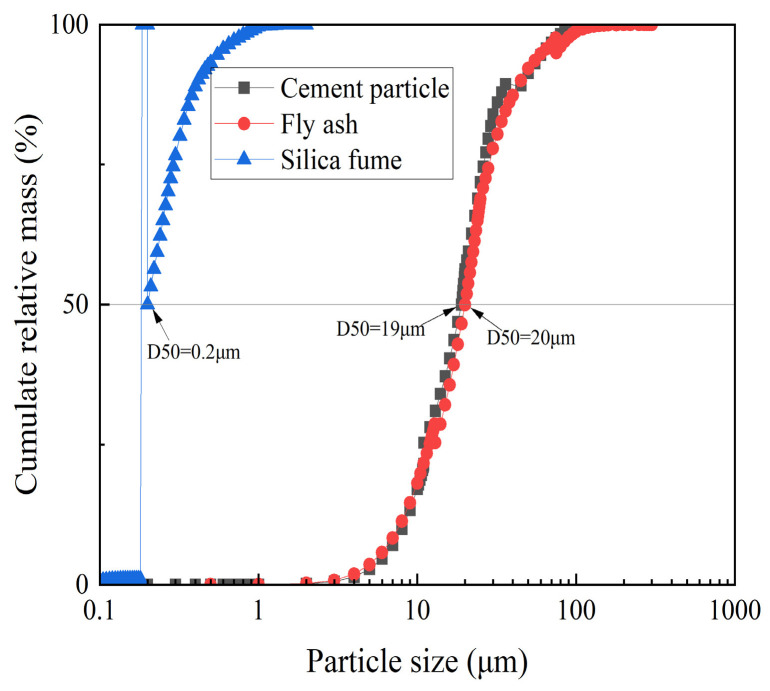
Particle size distribution of cement, fly ash, and silica fume.

The materials used in this study included P·O 42.5R cement (classified as CEM II under EN 197-1:2011) [10], it was selected for its balanced early-strength development (24 h compressive strength ≥ 20 MPa) and compatibility with pressing–dewatering processes. Grade II fly ash was added to improve workability and long-term strength via pozzolanic reactions; silica fume is incorporated to densify the matrix by filling nano-scale voids between cement grains; its high SiO_2_ content (96.85%, Table 1) and specific surface area (15–25 m^2^/g) promote secondary hydration, and it comes from Henan Dezhu New Materials Technology Co., Ltd., Zhengzhou City, Henan, China. Also, the particle size distribution is given in Figure 4. The aggregate consisted of natural river sand, including fine sand (0.5–1.2 mm) and coarse sand (1.2–2.4 mm), with a continuous grading and a density of 2650 kg/m^3^. Also, the polycarboxylic acid water-reducing agent with a water reduction rate exceeding 30% was selected for its high dispersion efficiency and adaptability to low water–cement ratios. The viscosity-reducing agent from Shanghai De Wei Nuo New Material Technology Co., Ltd., Chongming District, Shanghai, China, was used to improve the rheology of mortar. The chemical compositions of the cement, fly ash, and silica fume are shown in Table 1. Conventional production ratios for the pressed and dewatered cement products are shown in Table 2. The design strength classes are C40, C50, and C60.

### 2.2. Compression Dewatering Forming Process

**Figure 5:**
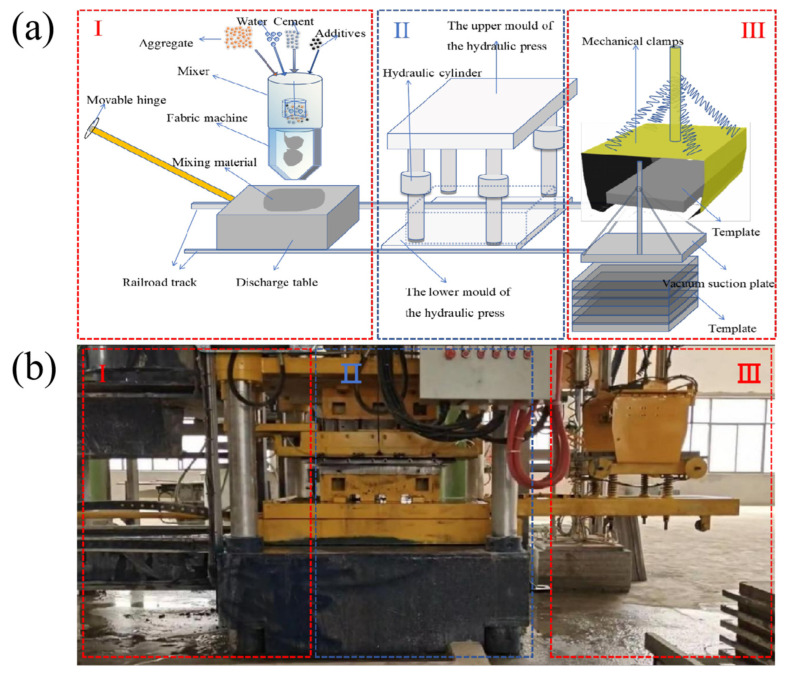
(**a**) Compression process flow diagram. (**b**) Compression process equipment diagram.

The compression dewatering forming process is shown in this chapter, and the specific flow of the process is as follows. The compression and dewatering forming is distributed in 5 steps (Figure 5), which included mixing, feeding (area I), compression, dewatering and forming (area II), and yarding (area III). First, the weighed materials (cement, sand, water, external admixtures, and additives) were placed into a vertical shaft planetary mixer. The mixer is designed to eliminate dead zones, with its mixing arms covering the bottom plate every 5 s. Once mixing was complete, the material was transferred to the fabric machine, which used the volumetric dosing method to dispense the mixed materials onto the discharge table (I area). Subsequently, the materials were placed on the discharge table (I area), and the discharge table was moved to the lower mold of the hydraulic press, which was uniformly equipped with drainage holes to ensure smooth drainage. The upper mold of the hydraulic press was located on the hydraulic cylinder, featuring a modular design on the surface of the hydraulic cylinder. The upper mold quickly compressed the mixture, filling the entire mold cavity instantly (II area). At the same time, a holding pressure of 10 MPa was applied for approximately 10 s, causing the excess water inside the mixing material to be pressed out. As a result, the products were shaped. After compression, the discharge table was pushed to the vacuum suction plate placed in advance on the template. Finally, the template was moved to the designated position (III area) using mechanical clamps. The overall compression process flow diagram is shown in Figure 5a, and the equipment diagram used is shown in Figure 5b.

The entire process offered several advantages, namely a high production efficiency, where a single piece had a forming time of 15~30 s; high product precision, due to the use of steel mold compression, where the general precision accuracy was within 1 mm; and the final products had a more delicate appearance compared to dry compression.

## 3. Results and Discussion

### 3.1. Experiments on the Rheological Properties

**Figure 6:**
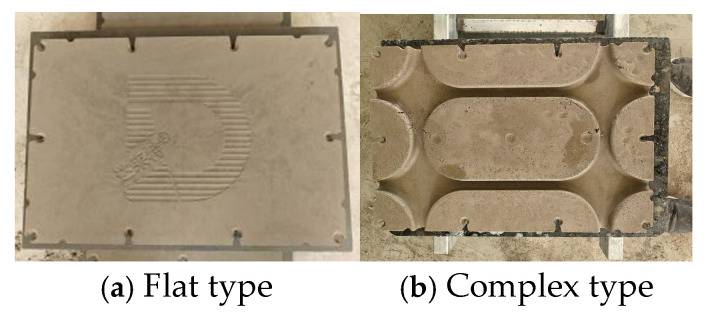
Pressed cement modular products.

The application of conventional production ratios (Table 2) in cement product manufacturing enabled the effective forming of the flat-type (Figure 6a), as evidenced by compaction process validation studies. However, challenge arises when attempting to press cement products with complex shapes. This resulted in significant material gaps and surface defects, such as pockmarks, as shown in Figure 6b. We attribute this result to the insufficient fluidity of mixing materials, also referred to as mortar in the text. In order to press complex-shaped cement products with fewer defects, the rheological properties of the pressed and dehydrated mortar were investigated and improved in this section.

#### 3.1.1. Rheological Test Method Selection

**Figure 7:**
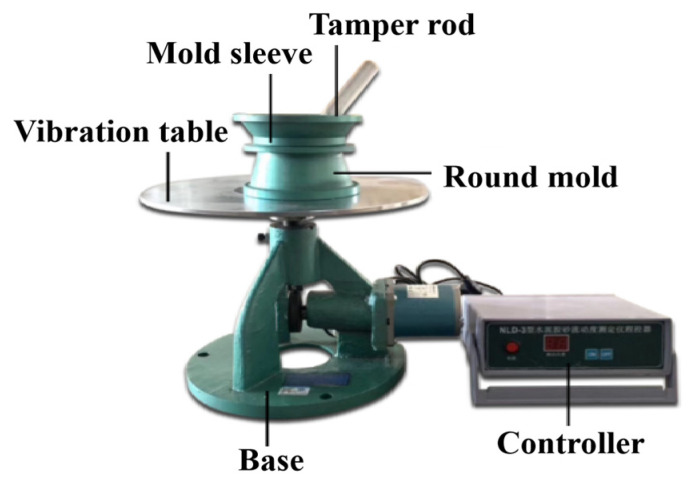
Flow table test instrument.

The pressed dehydrated cement material was plastic after mixing and then added to the mold for compression and forming. Two traditional experimental methods were used to characterize the rheology of the material. (1) The consistency of the mortar was tested according to the JGJ/T70-2009 standard to test the basic properties of building mortars [11]. (2) The fluidity of the mortar was measured based on a flow table test (the instrument as shown in Figure 7), according to the Chinese standard GBT-2419-2005 [12], which evaluates the spread diameter of fresh mortar after vibration on a flow table.

The rheological properties of the mortar prepared with conventional proportions (Table 2) were initially evaluated using standardized methods. First, the consistency was measured according to the JGJ/T70-2009 standard. The results (Table 3) revealed an extremely low consistency value of 11-14 mm, far below the detectable range (30–100 mm) specified by RISN-TG008-2010 [13] for common building mortars. Second, the fluidity was assessed via the flow table test (GB/T 2419-2005). Despite 25 controlled vibrations on the flow table, the mortar exhibited a maximum spread diameter of 118 mm (initial mold diameter:100 ± 0.5 mm), corresponding to a mere 13–18 mm expansion in any radial direction. This value is critically lower than the typical threshold for fluid mortars, indicating negligible flowability under standard test conditions. These quantitative results (consistency = 14 mm, spread diameter = 118 mm) conclusively demonstrate that traditional flow-based methods are unsuitable for characterizing such low-fluidity mixtures. Due to the limitations of flow table tests, we employed a modular compact mobile rheometer (MCMR) to measure the yield stress and plastic viscosity, which are critical parameters for low-fluidity mortars. This approach provided a direct rheological characterization beyond the resolution of conventional flow-based methods.

**Figure 8:**
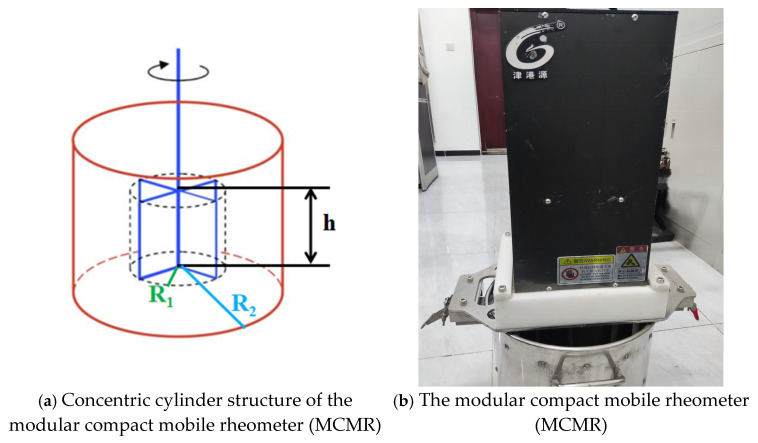
Concentric cylinder structure of the MCMR and the MCMR.

The rheological characterization of the processed mortar was conducted using the modular compact mobile rheometer (MCMR), which comes from the Jin Gang Yuan Instrument Company, enabling a precise measurement of the flow properties under controlled experimental conditions. The radius of the rheometer’s test drum was 140 mm, with a blade height of 125 mm and diameter of 125 mm. The freshly mixed cement material was loaded into the test drum of the rheometer, and the blade was rotated by an electric motor (Figure 8), with the rotational speed set to 0.025 rps to obtain the stress growth curve. The maximal torque obtained during the course of the test corresponded to the static yield stress of the material, which indicated that the fresh mortar had to transition from a static state to the point where it started to flow, to overcome the stress.

**Figure 9:**
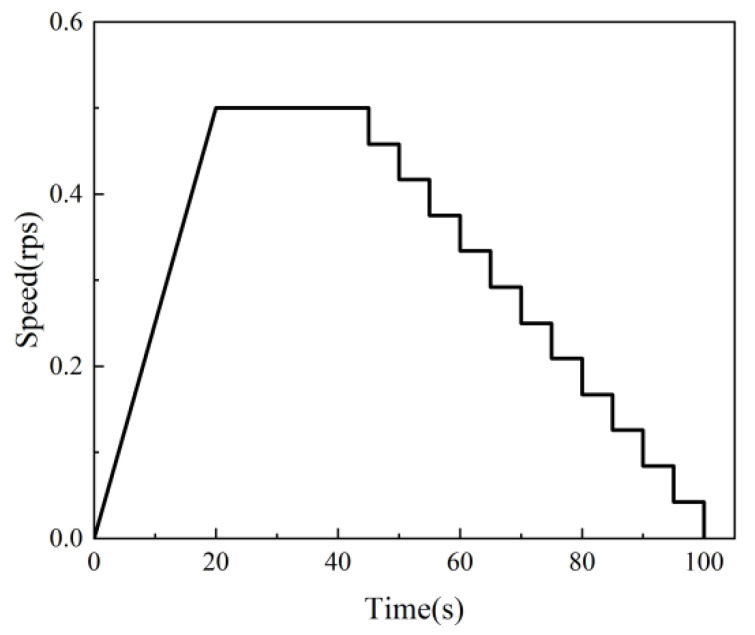
The rotational speed as a function of time.

The fresh mortar was then poured out, remixed, and loaded into the test kettle. The rotation speed was set to increase uniformly to 0.5 rps after 20 s and held for 20 s as the pre-shear stage, and then, the rotational speed was reduced step-by-step and held for 5 s at each stage. The rotational speed as a function of time is shown in Figure 9. The torque and rotational speed were measured by the rheometer in real time, and the torque (T) and rotation speed (N) were obtained for each group of steady flow conditions. The linear relationship between the torque and rotational speed was obtained by fitting according to the measured data [14] and the intercept G and slope H, as shown in Equation (1). The rheology of the freshly mixed cement mortar was described by the Bingham model [15], as shown in Equation (2). According to the Reiner–Riwlin equation [16,17], shown in Equation (3), the dynamic yield stress and plastic viscosity characterizing the fresh mortar could be calculated from the values of G and H, respectively:

*T* = *G* + HN, (1)

τ = τ_0_ + μ, (2)

(3) $$\tau_{0}=\frac{\left(\frac{1}{{R_{1}}^{2}}-\frac{1}{{R_{2}}^{2}}\right)}{4\pi h\ln\left(\frac{R_{2}}{R_{1}}\right)}G\;\mu =\frac{\left(\frac{1}{{R_{1}}^{2}}-\frac{1}{{R_{2}}^{2}}\right)}{8\pi^{2}h}H,$$

where *T* denotes the torque (N/m), the rotational force resisting flow, directly related to material yield stresses; N is the speed of rotation (rps), influencing viscosity calculations; τ_0_ is the dynamic yield stress (Pa), minimum stress to sustain flow, empirically derived from torque–rotation speed curves; μ is the plastic viscosity (Pa·s), reflecting resistance to shear deformation, slope of the linear region of the flow curve; *h* is the height of the blade (m); *R*_1_ denotes the blade radius (m); and *R*_2_ is the radius of test drum (m).

The modular compact mobile rheometer (MCMR) was used to test the mortar according to the conventional production ratios, with the test data shown in Table 4. The size of the peak torque was measured, and the static and dynamic yield stress and plastic viscosity were obtained according to Equations (1)–(3). The static yield stress indicated that the stress that the cement material fresh mortar had to overcome was from the static state to the beginning of the flow, reflecting the ability of the cement material to resist its own gravity or external load without deformation when at rest. The dynamic yield stress indicated the minimum stress required to maintain the flow of the fresh mortar, reflecting the ability of the cement material to flow under the action of an external load. The plastic viscosity reflected the relationship between the action of the stress and the flow speed of the fresh mortar. Meanwhile, the plastic viscosity reflected the relationship between the applied stress and the flow rate of the fresh mortar. These data more accurately characterized the rheological properties of the materials compared to traditional test methods. Therefore, subsequent experiments used the rheometer as the main test method for rheological properties.

#### 3.1.2. Effect of Mortar Design Ratios on Rheological Properties

To further investigate the influencing factors on the forming of dehydrated mortar and their mechanisms, the ratios of fly ash, silica fume, water reducer, and viscosity reducer were set as variables, as shown in Table 5. Specifically, 1a–1c consisted of fly ash mixed with 10–30% (mass fraction) alone, while groups 2a–2e consisted of fly ash mixed with 10–30% (mass fraction) fly ash and 10–30% (mass fraction) silica fume. Finally, groups 3a–3c and 4a–4e consisted of water reducing agent and viscosity reducing agent mixed according to the manufacturer’s specifications.

(1) Effect of fly ash on rheology

**Figure 10:**
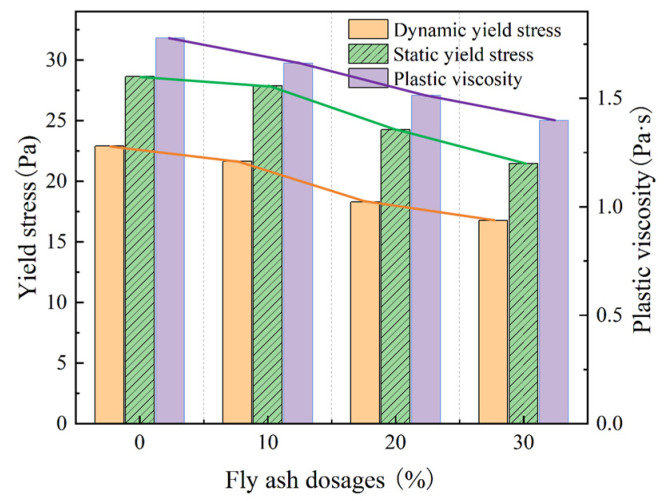
Rheological properties of the fresh mortar with different fly ash dosages.

To analyze the effect of different dosages of mineral admixtures on the rheological properties of the fresh mortar, experimental groups 1a–1c were used to conduct the experiments, using 10%, 20%, and 30% fly ash to replace the cement. The obtained experimental results are shown in Figure 10.

As shown in Figure 10, a linear regression analysis was performed on the rheological parameters of the mortar (static and dynamic yield stresses and plastic viscosity) as a function of the fly ash dosage. The results indicated a strong linear relationship, with R^2^ values of 0.96 for the static yield stress, 0.95 for the dynamic yield stress, and 0.99 for the plastic viscosity. As the dosage of fly ash increased, the static yield stress (i.e., the fresh mortar had to overcome lower stress at the beginning of the flow) decreased linearly. Similar linear trends were observed for the dynamic yield stress and plastic viscosity. When the dosage of fly ash was 30%, the static yield stress of the fresh mortar was 21.471 Pa, the dynamic yield stress was 16.764 Pa, and the plastic viscosity was 1.398 Pa·s. This was the lowest value in this experimental group, indicating that with an increase in fly ash, the fresh mortar improved and could easily flow. Therefore, the mix could quickly fill in the cavities of the formwork during the process of compaction.

First, the appearance of fly ash and silica fume particles was similar to the shape of a sphere, resulting in the ball effect [18,19], where under the action of shear force, the fly ash and sand particles could easily slide past each other. Therefore, the degree of bonding between the particles was reduced, and the structure could easily disintegrate. Secondly, due to its large specific surface area, it could adsorb some of the free water. During cement hydration, the adsorbed water was gradually released, increasing the water content in the fresh mortar. As a result, the continuous frictional resistance between the particles and resistance to flow was reduced [20]. The torque required to maintain the flow of the fresh mortar was smaller, and the static and dynamic yield stress value and plastic viscosity were reduced.

(2) Effect of fly ash (FA) and silica fume (SF) compounding

**Figure 11:**
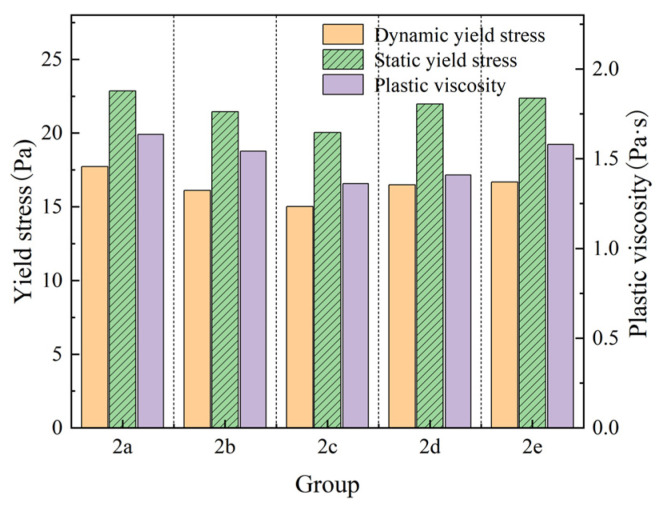
Rheology of slurries with different compounding ratios.

The supplementary cementitious materials (FA + SF) ratio was fixed at 0.7:0.3 (by weight) based on preliminary optimization (Experiment 1a–1c), ensuring a balance between workability and strength. Within this supplementary cementitious materials ratio (0.7:0.3), experimental groups 2a–2e were set up with a systematic variation in the ratio of FA:SF from 25:5 to 5:25 (experimental group 2a 25:5; group 2b 20:10; group 2c 15:15 group 2d 10:20; group 2e 5:25), to investigate their synergistic effects on rheology. The experimental results are shown in Figure 11.

As shown in Figure 11, after increasing the proportion of silica fume, the yield stress and plastic viscosity of the mixture were reduced. However, when the dosage of silica fume was greater than 15% (experimental group 2c, the dosage of SF = 15%; experimental group 2d, the dosage of SF = 20%; experimental group 2e, the dosage of SF = 25%), the static and dynamic yield stress increased and the rheology of the mixture started to deteriorate. This was because when the silica fume dosage was less than 15%, the ball effect of the particles in the composite cement material system was more obvious, the lubrication effect increased, and the fresh mortar fluidity increased. When the dosage of silica fume was greater than 15%, because the silica fume particles were finer than the fly ash particle size, they could fully fill the voids between the hydrated particles, greatly increasing the density of the particle system. At this point, the surface adsorption effect of the small particles masked the ball effect, and particle hydration required more water, increasing adhesion between the gel particles [21,22,23]. The experimental data showed that the fresh mortar with the lowest stress, promoting efficient flow, was achieved in experimental group 2c. This group allowed the fresh mortar to fill the template cavity with the lowest stress quickly. Thus, experimental group 2c had a more desirable rheology, and the cement material had a cement:fly ash:silica fume ratio of 0.7:0.15:0.15. The static yield stress of the fresh mortar was 20.042 Pa, while the dynamic yield stress was 15.012 Pa, and the plastic viscosity was 1.361 Pa·s. Therefore, the best rheology of the fresh mortar was achieved with the addition of 15% fly ash and silica fume.

(3) Influence of water reducing agents

**Figure 12:**
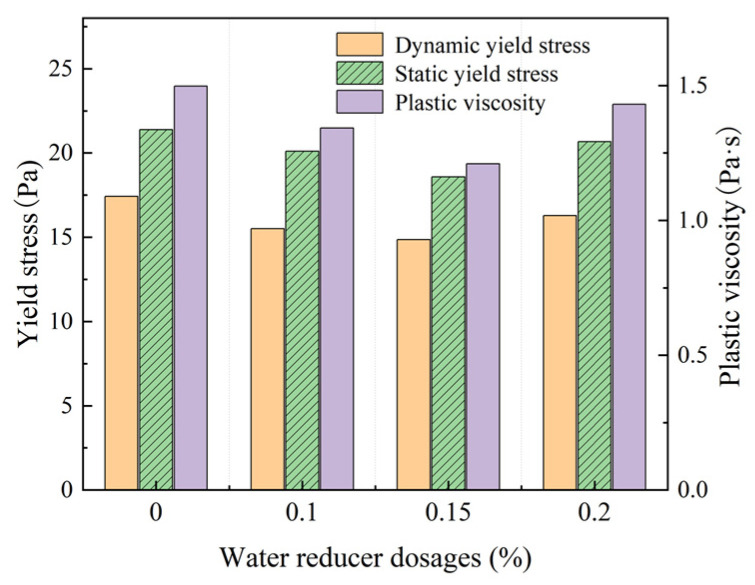
Rheology properties of fresh mortar with different water reducer dosages.

The rheological properties of the fresh mortar obtained through experimental groups 3a–3c are shown in Figure 12.

As shown in Figure 12, the water-reducing agent effectively reduced the static and dynamic yield stress and plastic viscosity of the fresh mortar and improved the rheology of the fresh mortar. This was due to the water-reducing agent, the cement particles on the surface with the same charge, and the formation of electrostatic repulsion. This caused the cement particles to disperse, allowing 10–30% of the free water to be released, effectively increasing the fluidity of the fresh mortar [24]. The water-reducing agent had strong hydrophilic groups, which enabled the formation of a stable solvated water film on the surface of cement particles, in which water molecules were incorporated. This water film provided excellent lubrication, reducing friction between cement particles. As a result, the rheological properties of the fresh mortar improved. Specifically, the lubricating effect of the solvated water film reduced the sliding resistance between the particles, enhancing the fluidity of the fresh mortar [25,26,27]. This resulted in a decrease in torque of the initial flow of the fresh mortar, where the static yield stress decreased. At the same time, this reduced the resistance to the flow of the interior of the fresh mortar and the torque of the interior of the fresh mortar, to maintain the flow of the fresh mortar, was reduced, i.e., the dynamic yield stress was reduced. As shown in Figure 12, the static yield stress of the fresh mortar was lower than 21.000 Pa and the dynamic yield stress was lower than 17.000 Pa after the water reducing agent was added.

(4) Effect of viscosity-reducing agents

**Figure 13:**
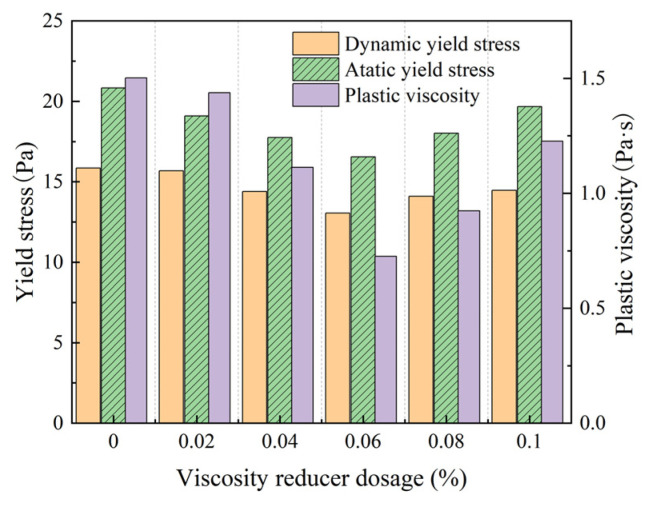
Rheology of fresh mortar with different dosages of viscosity reducers.

The effect of a viscosity reducer on the rheology of the fresh mortar was obtained from experimental groups 4a–4e, with the results shown in Figure 13.

As shown in Figure 13, the static and dynamic yield force of the fresh mortar decreased with an increasing viscosity-reducer dosage. When the dosage of the viscosity reducer was 0.06%, the static and dynamic yield stresses were the lowest, with values of 16.541 and 13.053 Pa, respectively. The plastic viscosity of the fresh mortar was 0.726 Pa·s, which demonstrated a decreasing trend. As the viscosity-reducing agent used in this experiment was polymerized from a variety of compounds with different molecular weights, the polymerization process formed many unique functional groups, which increased the chain length of the polymer. This effectively increased the reaction space of the granular system, and the viscosity-reducing agent formed a layer of strong dispersants in the hydrated granular system, which occupied the voids in the system and reduced the internal friction in the fresh mortar, reducing the viscosity of the fresh mortar [28,29]. However, when the dosage of the viscosity-reducing agent was greater than 0.06%, the effect of the viscosity-reducing agent was reduced, and the yield stress of the fresh mortar was increased to a certain extent. This was because the viscosity-reducing agent contained polyethylene glycol and other hydrophilic substances with high hydrophilicity [30]. When the content was increased, the thickness of the water film layer on the surface of the cement particles increased, binding more free water, reducing the viscosity, and even reducing the fluidity of the fresh mortar. Therefore, the dosage of the viscosity reducer should be less than 0.06%.

(5) Synthesis of Experimental Results

Integrating the optimal mix proportion of fly ash, silica fume, water reducing agent, viscosity reducing agent and their corresponding rheological parameters, this study reveals the influencing law of a mineral admixture and additives on the rheological performance of mortar through systematic experiments:

- When the dosage is less than 30%, fly ash significantly reduces the yield stress and plastic viscosity through the “ball bearing effect”, improving fluidity of the fresh mortar.
- The best rheology of the fresh mortar was achieved with the addition of 15% fly ash and silica fume due to the filling effect.
- The recommended mix proportion is 15% fly ash, 15% silica fume, 1.5% water-reducing agent, and 0.06% viscosity-reducing agent, which can effectively reduce surface defects and support the efficient production of complex shaped precast components, such as heated suspended floors.

### 3.2. Study of the Relationship Between Rheology and Formability

Based on the research conducted in Section 3.1.2 on the rheological properties of pressed dehydrated mortar, a proportioning system suitable for the pressed dehydrated modeling of complex-shaped cement products was developed.

To investigate the relationship between the rheological properties and formability of mortars, complex-shaped cement products were fabricated through a controlled pressing process using mortars with varying ratios. The formability was systematically evaluated by characterizing surface defects defined as follows:

- Lack of material incompleteness: Visible voids or missing regions resulting from insufficient mold filling.
- Surface cracking: Irregular tortoiseshell-like fracture patterns.
- Surface pitting: Localized roughness and micro-depressions.

**Figure 14:**
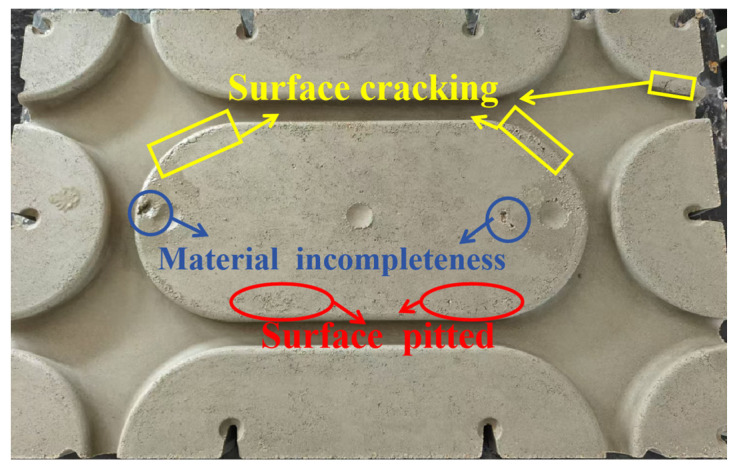
Classification of defects in fabricated products.

These defect categories (illustrated in Figure 14) served as quantitative indicators of formability. Rheological parameters, including the yield stress and plastic viscosity, were subsequently correlated with defect occurrence rates through statistical analysis to establish predictive criteria for optimal mortar formulations.

Based on the quantitative criteria of the defect count and area, product quality was categorized into three grades: Grade A, Grade B, and Grade C. When the total number of defects did not exceed 2 and the total area of defects did not exceed 5 cm^2^, the product was classified as Grade A. When the total number of defects did not exceed 4 and the total area of defects did not exceed 10 cm^2^, it was classified as Grade B. When the total number of defects did not exceed 6 and the total area of defects did not exceed 20 cm^2^, it was classified as Grade C. The defect count and area of each product were measured to assign its quality grade under each mortar formulation. Rheological properties of the formulations were then statistically correlated with the quality grades, as summarized in Table 6.

According to the analysis conducted in Section 3.1.2 and Table 6, the reduction in static and dynamic yield stress had a significant effect on the forming of products. When the static yield stress was greater than 24.000 Pa and the dynamic yield stress was greater than 19.000 Pa, the apparent performance of complex-shaped cement products belonged to Grade C and the products were unusable. When the static yield stress was 21.000 ≤ τ_0_ ≤ 24.000 and the dynamic yield stress was 16.000 ≤ τ ≤ 19.000, the apparent properties of the complex molded cement products belonged to Grade B and the quality of the products was barely acceptable. Only with a static yield stress τ_0_ < 21.000 and dynamic yield stress τ < 16.000 did the apparent properties of the complex molded cement products belong to the Grade A category, allowing the resulting products to be used. In this experiment, no products defined as excellent were produced, as all the products had some defects, possibly related to the limitations of the process itself. However, the fabricated products could meet the requirements for use.

### 3.3. Effect of Compression Dewatering on the Compressive Strength of Mortar

#### 3.3.1. Experimental Method for Compressive Strength

**Figure 15:**
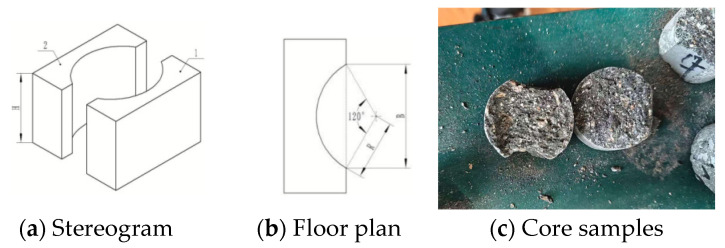
Arc-face pressure test setup [31].

Due to the uniqueness of the compression and dewatering forming process, which made it difficult to produce standard specimens for mechanical property testing, in this study, the Technical Standard for Testing the Compressive Strength of Cement-based Materials by the Arc-Face Pressure Method, DB13(J)/T 8533-2023 [31], was used. First, using the drilling core sampling method, core samples were drilled with a diameter of 38 mm and the minimum length of 61 m in the pressed and dehydrated molded cement products, as shown in Figure 15c. An arc-face compression apparatus, as shown in Figure 15, Figure 15a is its stereogram and Figure 15b is its floor plan, was employed to determine the compressive strength of mortar based on the peak destructive load value. The load was applied to the arc-face compression specimen, and the compressive strength of the mortar was calculated by measuring the maximum load at failure.

#### 3.3.2. Effect of Press Dewatering on Compressive Strength

In this study, three conventional production ratios of C40, C50, and C60 were used to produce pressed (pressure of 8 MPa) and unpressed dehydrated specimens, as shown in Table 7, for a comparison.

As shown in Table 7, compression dewatering and forming significantly increased the strength of the specimens relative to specimens not subjected to compression forming. The C40 material demonstrated an increase in strength by an average of 21%. As the strength grade increased, the proportion of strength improvement by press dewatering decreased, including 16% for C50 and 11% for C60.

Overall the compressive strength of the cement products could be increased more substantially through compression and dewatering forming, mainly because compression and dewatering reduced the water–cement ratio and porosity of the material. To further elucidate the mechanisms underlying the strength enhancement, the microscopic morphology of the material was analyzed, revealing the structural contributions to the improved mechanical performance.

### 3.4. Mechanism of Micro-Action of Compression Dewatering on Mortar

The compression dewatering process reduced the water in the mortar by about 20%, which positively affected the strength. At a deeper level, the mechanism of compression dewatering reduced the water while decreasing the porosity of the cement products, thus increasing their strength. In this study, low-field nuclear magnetic resonance (NMR) was used to examine the effect of different pressures on water migration and the porosity of cement products after compression dewatering. The relationship between compression dewatering and the strength enhancement of cement products was further analyzed.

#### 3.4.1. Test Methods of Low Field Nuclear Magnetic Resonance

**Figure 16:**
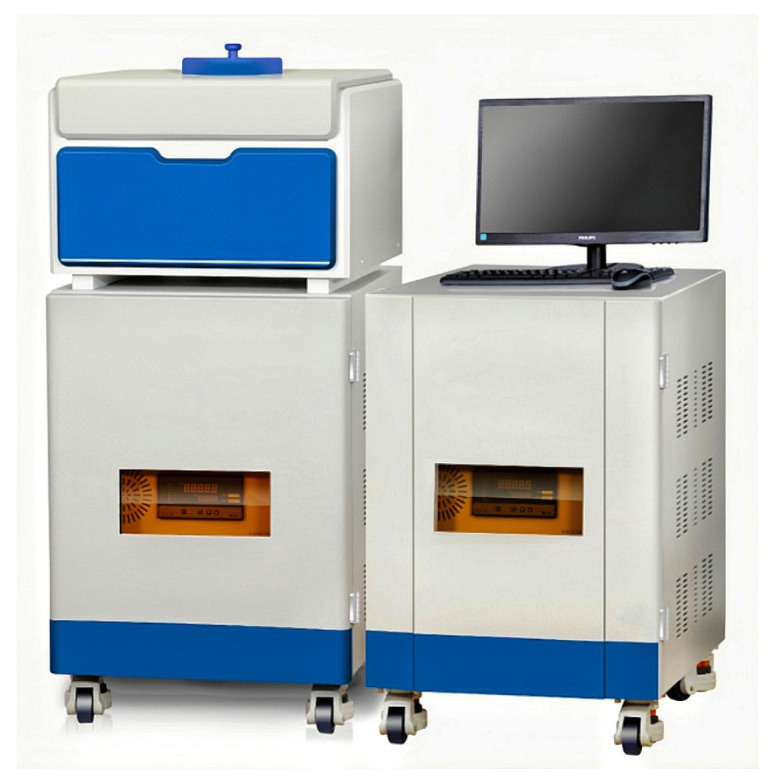
Low-field nuclear magnetic resonance experimental apparatus.

The low-field NMR technique used water molecules as probes to detect the relaxation signals of hydrogen protons [32], with the device shown in Figure 16. The transverse relaxation time served as a characteristic quantity that characterized how fast or slow the transverse magnetization vector returned to equilibrium, capable of reflecting the water degree of freedom and the phase distribution inside the samples. The length of relaxation time reflected the degree of freedom of moisture inside the module, where the shorter the relaxation time, the lower the degree of freedom of moisture, and the longer the relaxation time, the higher the degree of freedom. The peaks in the transverse relaxation time relaxation spectra represented the different moisture phases inside the samples. An analysis of the relaxation time and signal amplitude from the transverse relaxation time inversion spectra enabled an investigation of moisture content changes within the module and their migration patterns.

#### 3.4.2. Effect of Press Dewatering on Moisture Migration

**Figure 17:**
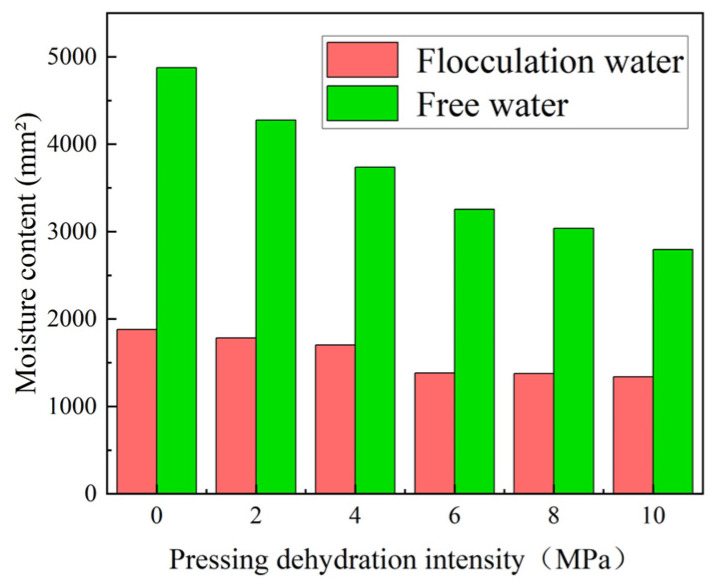
Relationship between moisture content and pressing dehydration intensity.

The initially hydrated mortar was still in a plastic flowable state with a low degree of hydration reaction, and the mortar could be simplified to a suspension system composed of water and solid particles. In the cement paste, water primarily occupied interstitial spaces between particles and could be categorized as free water (capillary pores > 50 nm) or flocculated water (physically adsorbed on particle surfaces) [33]. In this study, pressures of 2, 4, 6, 8, and 10 MPa were used to press and dehydrate the mortar, and to study the effect of compression and dewatering on the form, content, and proportion of water inside the mortar. The effect of compression and dewatering on the microscopic properties was analyzed in detail. The experimental results are shown in Figure 17, indicating that the contents of the two types of water in the mix decreased with pressure; however, the reduction rate was not the same.

Free water, also termed unbound water, primarily resides in capillary pores (>50 nm) within the cement matrix and exhibits weak physical interactions with the solid phases, rendering it unstable and susceptible to evaporation or migration under external forces. In this study, its decreasing trend was the most obvious, with the water content decaying at a rate of 10%, and the final water loss was about 43% by 10 MPa. However, the de-creasing trend slowed down. The large reduction in this type of water after compression was the main reason for the setting of cement products.

Flocculating water refers to water trapped within the flocculating structure of cement particles, which occurred due to the fine particles in the cement powder. This water was greatly influenced by the surface, resulting in a short relaxation time, controlled within 1.5 ms. Under pressure, the amount of flocculating water decreased, while at 6 MPa, it gradually leveled off, after which the water loss stabilized. The final loss of flocculating water was approximately 29%. The reduction of flocculating water contributed positively to the shaping and strength of the cement products.

#### 3.4.3. Effect of Compression Dewatering on the Porosity of Mortar

In this study, NMR was used to test the moisture of cement mortar in a saturated water state. Because the mortar was in a saturated water state, the volume of moisture was considered the volume of mortar porosity, and the change in porosity of the mortar was tested under pressures of 2, 4, 6, 8, and 10 MPa.

Due to the overall low degree of the hydration reaction, only some of the mortar could easily hydrolyze and reactive minerals, such as C_3_A, reacted. As a result, a specific pore structure did not form, and the internal pore basically consisted of pores between the particles and pore throat (pore linkage between the pore channels).

**Figure 18:**
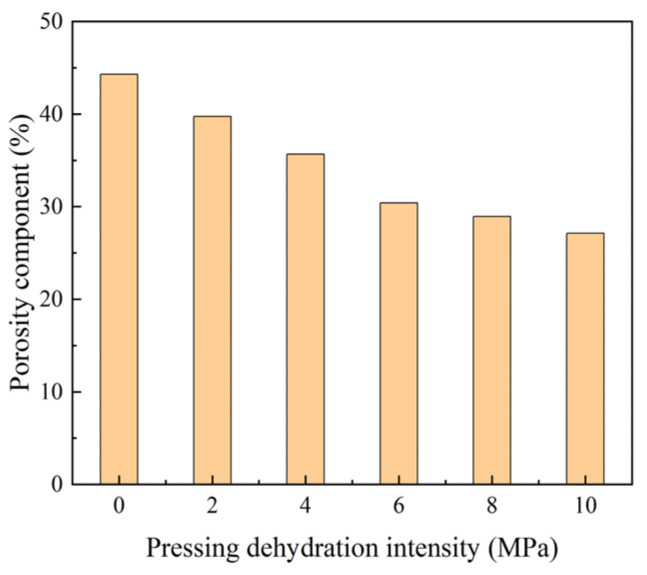
Porosity of mortar at different pressures.

As shown in Figure 18, the porosity content decreased rapidly with an increasing pressure, and the initial porosity content was 44.3%. After reaching 6 MPa, it tended to stabilize, and the decreasing trend was slightly slower, with final porosity content of 27.1%, resulting in a reduction of 38.8%. This showed that the water was discharged under pressure, and the cement particles were squeezed, occupying the original pore position, and the cement particles in the same section were more uniformly and densely distributed. Thus, the degree of concrete filling was higher per unit volume, and the concrete strength and seepage resistance were effectively improved.

#### 3.4.4. Effect of Compression Dewatering on the Pore Structure of Hardened Mortar

The above analyses and studies of water migration, as well as the porosity of unreacted cement-based mortar, revealed the effect of compression dewatering on the microscopic properties of cement-based mortar. To understand the effect of compression dewatering on the porosity of the hardened final cement products, in this study, the microscopic morphology of the C50 hardened mortar (28 days) after compression dewatering (unpressurized) and 8 MPa of compression was examined via scanning electron microscopy (S-4800, from Hitachi, Tokyo, Japan).

**Figure 19:**
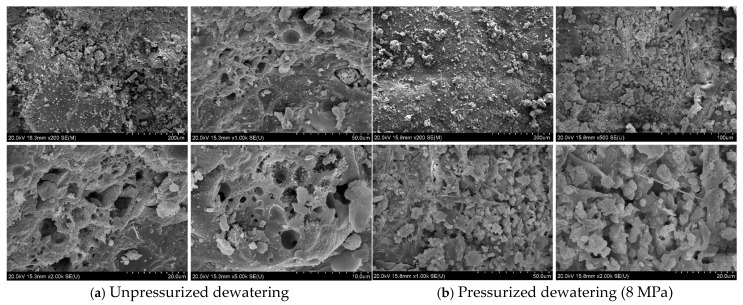
Microscopic morphology of hardened mortar (28 days).

As shown in Figure 19, the pore structure was observed, respectively, at 10, 20, 50, 100, and 200 μm. Before compression, the pore shape was roughly circular, and the diameter sizes differed, with pore sizes of 1–50 μm, and the gap in the shape of gel particles was obvious in the cross-section. After compression, the material showed various irregular pore structures, such as rods and ellipsoids, with no large pores and a uniform size, and the diameter of the pores was between 0.5 and 20 μm. The content of gel particles obviously increased, verifying that gel particles occupied the pore space of the pressed water, and the compactness increased accordingly.

**Figure 20:**
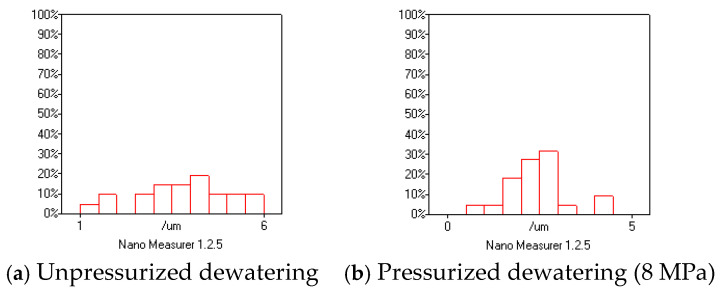
Histogram showing the porosity of the hardened mortar (28 days).

Figure 20 presents the porosity distribution graph obtained via electron microscopy, indicating that the maximum pore size before compression was 5.93 μm, and the average pore size was 3.84 μm, with pores less than 3.00 μm occupying 23.8% of the total pores. After compression, the maximum pore size was 4.27 μm, and the average pore size was 2.39 μm, with pores less than 3.00 μm occupying 86.4% of the total pores, with a change of 72.4%. Therefore, compression forming dewatering played an effective role in limiting the multiple harmful pores present in the hardened mortar.

#### 3.4.5. Analysis of the Relationship Between the Compressive Strength and Microscopic Properties of the Mortar

According to the above experiments, compression dewatering had a forming effect on the strength and microscopic properties of the cement products, which improved the pore structure and strength of the cement products. To further understand the relationship between the compressive strength and microscopic properties of the cement products in the states of compression and dewatering, the C50 material was used to test the effect of compression and dewatering on the strength and microscopic properties of mortar. Specifically, different pressures were used, with pressures of 2, 4, 6, 8, and 10 MPa. The microscopic properties included moisture migration and porosity, as the migration of moisture and porosity was difficult to directly describe using singular data. After the comparison, a more intuitive assessment of the mortar solid–liquid ratio was conducted to characterize the above parameters. Specifically, the mortar solid–liquid ratio was equal to the total volume of mortar solids divided by the total volume of water in the mortar.

**Figure 21:**
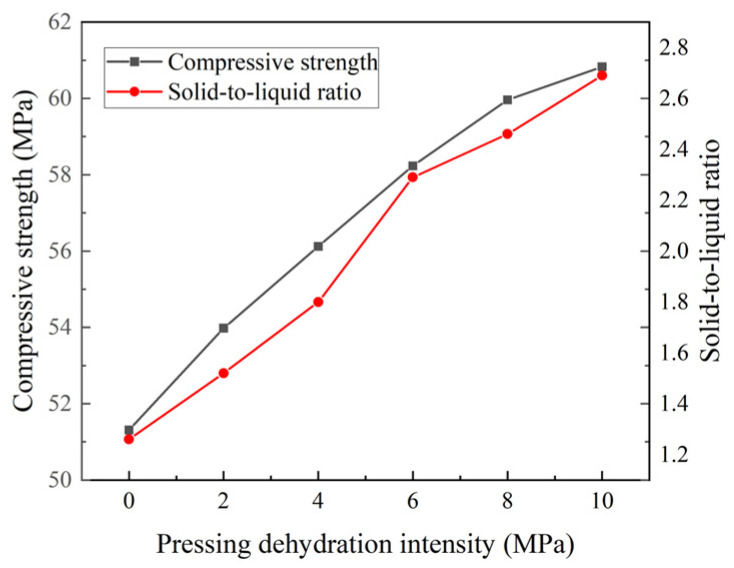
Relationship among compressive strength, solid-to-liquid ratio, and pressing dehydration intensity.

**Figure 22:**
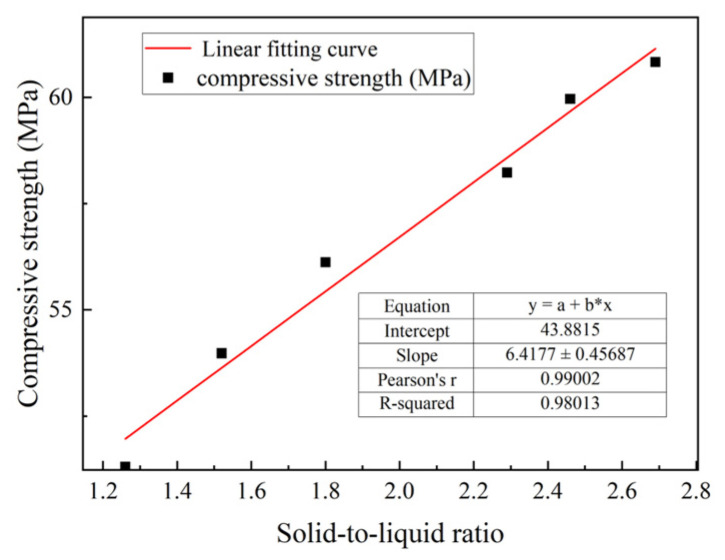
Fitted curve.

As shown in Figure 21, with an increase in the pressing dehydration intensity, the compressive strength of mortar and solid–liquid ratio gradually increased. However, the rate of increase gradually slowed, and the increase in pressing dehydration intensity from 8 to 10 MPa was limited to 1.45%. From an economic point of view, the optimal dewatering pressure was reached at 8 MPa. In addition, according to the linear fitting curve in Figure 22, the compressive strength and the solid-to-liquid ratio of the mortar (R^2^ was 0.98) were found to have relatively good consistency, because the solid-to-liquid ratio basically responded to the degree of densification of the mortar. This solid-to-liquid ratio was considered a prospective indicator of the compressive strength at a later stage.

## 4. Conclusions

This study explored a novel approach to produce high-strength and complex-shaped cement products utilizing the compression dewatering forming method. Experimental methods suitable for evaluating the fluidity and strength of mortar using a compression dewatering process were explored. This study also revealed the relationship between the rheology and formability of mortar, providing theoretical support for the suppressing production of complex-shaped cement products.

This compression dewatering forming method had an automatic loading and compression efficiency of up to 2000 pieces/day. In addition, this process used steel molds, and the precision error of the formed products was less than 1 mm. This compression and dewatering forming process, which effectively removed water and harmful pores, allowed for the use of molds with a low water–cement ratio to produce products with strengths greater than 70 MPa. This approach offers a novel solution for the high-efficiency production of complex-shaped cement products. To extend the application of the compression dewatering forming method, more in-depth research should be conducted in the future, for example, alternative industrial by-products (e.g., slag, recycled powder) for cost reduction, the compression dewatering effects of higher pressures (>10 MPa), and long-term durability research, etc. This study provides new ways for expanding the use of cement in applications, such as multifunctional prefabricated manhole covers, decorative components, cement vignettes, and even cement-based furniture. This process has the potential to significantly broaden the scope of cement product applications in the future.

## Figures and Tables

**Table 1:** Composition of cement, fly ash, and silica fume, %.

Project Material	CaO	SiO_2_	Al_2_O_3_	Fe_2_O_3_	MgO	SO_3_	K_2_O	Loss
Cement	57.71	23.43	7.93	2.94	3.16	2.09	-	2.74
Fly ash	4.85	54.46	26.56	5.16	0.46	1.24	3.48	3.79
Silica fume	0.23	96.85	0.41	-	0.33	-	0.23	1.95

**Table 2:** Conventional production ratios for the pressed and dewatered cement products.

Design Strength Class	Cement (g)	Fly Ash (g)	Fine Sand (g)	Coarse Sand (g)	Water (g)
C40	187	80	567	167	80
C50	233	100	500	167	100
C60	267	100	400	233	100

**Table 3:** Data from the standard test methods.

Design Strength Class	C40	C50	C60	Standard Requirement (mm)
Consistency (mm)	11	13	14	30–100
Extension (mm)	113	115	118	>180

**Table 4:** Modular compact mobile rheometer test data.

Design Strength Class	Peak Torque (N⋅m)	Static Yield Stress (Pa)	Dynamic Yield Stress (Pa)	Plastic Viscosity (Pa·s)
C40	77.07	24.565	19.684	1.734
C50	80.91	26.756	21.062	2.061
C60	86.61	29.584	25.931	2.398

**Table 5:** Mortar design ratios (wt%).

Experiment	Cement (g)	Sand (g)	Fly Ash (g)	Silica Fume (g)	Water (g)	Water Reducing Agent (%)	Viscosity Reducer (%)
0	1000	2000	0	0	380	0	0
1a	900	2000	100	0	380	0	0
1b	800	2000	200	0	380	0	0
1c	700	2000	300	0	380	0	0
2a	700	2000	250	50	380	0	0
2b	700	2000	200	100	380	0	0
2c	700	2000	150	150	380	0	0
2d	700	2000	100	200	380	0	0
2e	700	2000	50	250	380	0	0
3a	700	2000	100	200	380	1	0
3b	700	2000	100	200	380	1.5	0
3c	700	2000	100	200	380	2	0
4a	700	2000	100	200	380	0	0.02
4b	700	2000	100	200	380	0	0.04
4c	700	2000	100	200	380	0	0.06
4d	700	2000	100	200	380	0	0.08
4e	700	2000	100	200	380	0	0.1

**Table 6:** Relationship between rheological properties of materials and quality grade of products.

Quality Grade	Static Yield Stress (τ_0_)/Pa	Dynamic Yield Stress (τ)/Pa	The Total Number of Defects (t)	The Total Area of Defects (a)/mm^2^
Grade A	τ_0_ < 21.000	τ < 16.000	t ≤ 2	a ≤ 5
Grade B	21.000 ≤ τ_0_ ≤ 24.000	16.000 ≤ τ ≤ 19.000	t ≤ 4	a ≤ 10
Grade C	τ_0_ > 24.000	τ > 19.000	t ≤ 6	a ≤ 20

**Table 7:** Effect of compression dewatering on the compressive strength of mortar.

Design Strength Class	Compression and Dewatering Compressive Strength (MPa)	Unpressurised Dewatering Compressive Strength (MPa)	Percentage Increase in Intensity (%)	Average Percentage of Increase in Intensity (%)
C40	50.80	41.12	24	21
	52.21	42.30	23	
	51.02	43.93	16	
C50	60.49	50.29	20	16
	58.42	51.63	13	
	61.51	53.97	14	
C60	67.84	62.75	8	11
	70.21	61.63	14	
	71.31	63.47	12	

## Data Availability

The original contributions presented in this study are included in the article. Further inquiries can be directed to the corresponding author.
